# Supplementary material for: A rapid, low-cost, and highly sensitive SARS-CoV-2 diagnostic based on whole-genome sequencing
Source: PLoS One. 2023 Nov 30;18(11):e0294283. doi: 10.1371/journal.pone.0294283 (PMC10688730; doi:10.1371/journal.pone.0294283)
Supplement: S1 File — (PDF) [file pone.0294283.s006.pdf]

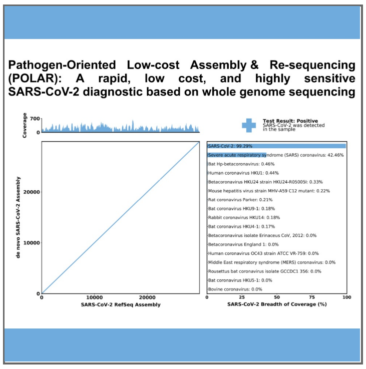

APR 25, 2020

Pathogen-Oriented Low-cost Assembly & Re-sequencing (POLAR): A highly sensitive and high-throughput SARS-CoV-2 diagnostic based on whole genome sequencing

Forked from nCoV-2019 sequencing protocol (single sample)

Per A. Adastra[1,2,3], Neva C. Durand[1,2,3,4], Namita Mitra[1,2,3], Saul Godinez[1,2,3], Ragini Mahajan[1,3,5], Alyssa Blackburn[1,2,3], Zane Colaric[1,2,3], Joshua W. M. Theisen[1,3], David Weisz[1,2,3], Olga Dudchenko[1,2,3], Andreas Gnirke[1,4], Suhas S.P. Rao[1,2,3,6], Parwinder Kaur[7], Erez Lieberman Aiden[1,2,3,8], Aviva Presser Aiden[1,2,9,10]

[1]The Center for Genome Architecture, Baylor College of Medicine, Houston, TX 77030, USA;
[2]Department of Molecular and Human Genetics, Baylor College of Medicine, Houston, TX;
[3]Center for Theoretical Biological Physics, Rice University, Houston, TX 77030, USA;
[4]Broad Institute of MIT and Harvard, Cambridge, MA 02139, USA ;
[5]B9Department of Biosciences, Rice University, Houston, TX 77030, USA;
[6]Department of Structural Biology, Stanford University School of Medicine, Stanford, CA 94305, USA;
[7]UWA School of Agriculture and Environment, The University of Western Australia, 35 Stirling Highway, Crawley, WA 6009, Australia;
[8]Departments of Computer Science and Computational and Applied Mathematics, Rice University, Houston, TX 77030, USA;
[9]Department of Bioengineering, Rice University, Houston, TX, USA;
[10]Department of Pediatrics, Stanford University School of Medicine, Stanford, CA 94305, USA

Erez Lieberman Aiden: Co-corresponding authors ;
Aviva Presser Aiden: Co-corresponding authors;

Coronavirus Method Development Community | Low-cost, high-quality ...

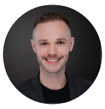

Per A. Adastra

OPEN ACCESS

DOI:
dx.doi.org/10.17504/protocols.io.bearjad6

Protocol Citation: Per A. Adastra, Neva C. Durand, Namita Mitra, Saul Godinez, Ragini Mahajan, Alyssa Blackburn, Zane Colaric, Joshua W. M. Theisen, David Weisz, Olga Dudchenko, Andreas Gnirke, Suhas S.P. Rao, Parwinder Kaur, Erez Lieberman Aiden, Aviva Presser Aiden 2020. Pathogen-Oriented Low-cost Assembly & Re-sequencing (POLAR): A highly sensitive and high-throughput SARS-CoV-2 diagnostic based on whole genome sequencing. **protocols.io** https://dx.doi.org/10.17504/protocols.io.bearjad6

License: This is an open access protocol distributed under the terms of the Creative Commons Attribution License, which permits unrestricted use, distribution, and reproduction in any medium, provided the original author and source are credited

Protocol status: Working
We use this protocol in our group and it is working, preprint coming out shortly.

Created: Mar 27, 2020

Last Modified: Apr 25, 2020

PROTOCOL integer ID: 34865

ABSTRACT

Here, we introduce a low-cost, high-throughput method for diagnosis of SARS-CoV-2 infection, dubbed Pathogen- Oriented Low-Cost Assembly & Re-Sequencing (POLAR), that enhances sensitivity by aiming to amplify the entire SARS-CoV-2 genome rather than targeting particular viral loci, as in typical RT- PCR assays. To achieve this goal, we combine a SARS-CoV-2 enrichment method developed by the ARTIC Network (https://artic.network/) with short-read DNA sequencing and *de novo* genome assembly. For details on our computational pipeline for automated data processing, including documentation and test set, please visit our Githuh at https://github.com/aidenlab/Polar.

GUIDELINES

SARS-CoV-2 Specific Primer Set

The ARTIC Network designed and tested[1] the primer set used in this protocol and must be custom ordered prior to experiments. Details on their primer set can be found on their Github page https://github.com/artic-network/artic-ncov2019.

1. Artic Network. https://artic.network/resources/ncov/ncov-amplicon-v3.pdf.

The World Health Organization: Dos and Don'ts for Molecular Testing (https://www.who.int/malaria/areas/diagnosis/molecular-testing-dos-donts/en/)

Molecular detection methods have the ability to produce a large volume of nucleic acid through the amplification of trace quantities found in samples. While this is beneficial for enabling sensitive detection, it also introduces the possibility of contamination through the spreading of amplicon aerosols in the laboratory environment. When conducting experiments, measures can be undertaken to avoid the contamination of reagents, laboratory equipment, and bench space, as such contamination may generate false-positive (or false-negative) results.
To help reduce the likelihood of contamination, Good Laboratory Practice should be exercised at all times. Specifically, precautions should be taken regarding the following points:

Handling reagents

- Briefly centrifuge reagent tubes before opening to avoid the generation of aerosols.
- Aliquot reagents to avoid multiple freeze-thaw and the contamination of master stocks.
- Clearly label and date all reagent and reaction tubes and maintain logs of reagent lot and batch numbers used in all experiments.
- Pipette all reagents and samples using filter tips. Prior to purchase, it is advisable

to confirm with the manufacturer that the filter tips fit the brand of the pipette to be used.

Organization of workspace and equipment

The workspace *should be organized to ensure that the flow of work occurs in one direction, from clean areas (pre-PCR) to dirty areas (post-PCR). The following general precautions will help to reduce the chance of contamination.*

Have separate designated rooms, or at minimum physically separate areas, for:
1. master mix preparation,
2. nucleic acid extraction and DNA template addition

In some settings, having 4 separate rooms is difficult. A possible but less desirable option is to do the master mix preparation in a containment area, e.g. a laminar flow cabinet. In the case of nested PCR amplification, the preparation of the master mix for the second round reaction should be prepared in the 'clean' area for master mix preparation, but the inoculation with the primary PCR product should be done in the amplification room, and if possible in a dedicated containment area (e.g. a laminar flow cabinet).

Each room/area needs a separate set of clearly labeled pipettes, filter tips, tube racks, vortexes, centrifuges (if relevant), pens, generic lab reagents, lab coats, and boxes of gloves that will remain at their respective workstations.
Hands must be washed and gloves and lab coats changed when moving between the designated areas. Reagents and equipment should not be moved from a dirty area to a clean area. Should an extreme case arise where a reagent or piece of equipment needs to be moved backward, it must first be decontaminated with 10% sodium hypochlorite, followed by a wipe down with sterile water

Ideally, staff should abide by the unidirectional workflow ethos and not go from dirty areas (post-PCR) back to clean areas (pre-PCR) on the same day. However, there may be occasions when this is unavoidable. When such occasion arises, personnel must take care to thoroughly wash hands, change gloves, use the designated lab coat and not introduce any equipment they will want to take out of the room again, such as lab books. Such control measures should be emphasized in staff training on molecular methods.

After use, bench spaces should be cleaned with 10% sodium hypochlorite (followed by sterile water to remove residual bleach), 70% ethanol, or a validated commercially available DNA-destroying decontaminant. Ideally, ultra-violet (UV) lamps should be fitted to enable decontamination by irradiation. However, the use of UV lamps should be restricted to closed working areas, e.g. safety cabinets, in order to limit

the laboratory staff's UV exposure. Please abide by manufacturer instructions for UV lamp care, ventilation, and cleaning in order to ensure that lamps remain effective.

If manufacturer instructions permit it, pipettes should be routinely sterilized by autoclave. If pipettes cannot be autoclaved, it should suffice to clean them with 10% sodium hypochlorite (followed by a thorough wipe down with sterile water) or with a commercial DNA-destroying decontaminant followed by UV exposure.

All equipment needs to be calibrated regularly according to the manufacturer-recommended schedule. A designated person should be in charge of ensuring that the calibration schedule is adhered to, detailed logs are maintained, and service labels are clearly displayed on equipment.

Use and cleaning advice for the designated molecular space

- **Pre-PCR: Reagent aliquoting / mastermix preparation**

This should be the cleanest of all spaces used for the preparation of molecular experiments and should ideally be a designated laminar flow cabinet equipped with a UV light.
Samples, extracted nucleic acid, and amplified PCR products must not be handled in this area.
Amplification reagents should be kept in a freezer (or refrigerator, as per manufacturer recommendations) in the same designated space, ideally next to the laminar flow cabinet or pre-PCR area.
Gloves should be changed each time upon entering the pre-PCR area or laminar flow cabinet.
The pre-PCR area or laminar flow cabinet should be cleaned before and after use as follows: Wipe down all items in the cabinet, e.g. pipettes, tip boxes, vortex, centrifuge, tube racks, pens, etc. with 70% ethanol or a commercial DNA-destroying decontaminant, and allow to dry. In the case of a closed working area, e.g. a laminar flow cabinet, expose the hood to UV light for 30 minutes.

- **Pre-PCR: Nucleic acid extraction/template addition**

Nucleic acid must be extracted and handled in a second designated area, using a separate set of pipettes, filter tips, tube racks, fresh gloves, lab coats, and other equipment.
This area is also for the addition of template, controls, and trendlines to the master mix tubes or plates. To avoid contamination of the extracted nucleic acid samples that are being analyzed, it is recommended to change gloves prior to handling positive controls or standards and to use a separate set of pipettes.
PCR reagents and amplified products must not be pipetted in this area.
Samples should be stored in designated fridges or freezers in the same area.

The sample workspace should be cleaned in the same way as the master mix space.

- **Post-PCR: Amplification and handling of the amplified product**

This designated space is for post-amplification processes and should be physically separate from the pre-PCR areas. It usually contains thermocyclers and real-time platforms, and ideally should have a laminar flow cabinet for adding the round 1 PCR product to the round 2 reaction, if nested PCR is being performed.
PCR reagents and extracted nucleic acid must not be handled in this area since the risk of contamination are high.
This area should have a separate set of gloves, lab coats, plate and tube racks, pipettes, filter tips, bins, and other equipment.
Tubes must be centrifuged before opening.
The sample workspace should be cleaned in the same way as the master mix space.

MATERIALS

MATERIALS

- Q5 Hot Start High-Fidelity DNA Polymerase - 100 units **New England Biolabs Catalog #M0493S**
- Deoxynucleotide Solution Set - 25 umol of each **New England Biolabs Catalog #N0446S**
- Random hexamers **Thermo Fisher Scientific Catalog #N8080127**
- Nextera XT DNA Sample Preparation Kit, 96 samples **illumina Catalog #FC-131-1096**
- Qubit dsDNA HS (High sensitivity) Assays **Thermo Fisher Scientific Catalog #Q32851**
- SuperScript™ IV Reverse Transcriptase **Thermo Fisher Scientific Catalog #18090050**
- RNaseOUT™ Recombinant Ribonuclease Inhibitor **Thermo Fisher Catalog #10777019**
- SuperPlate PCR Plate, 96-well, semi-skirted **Thermo Fisher Catalog #AB2400**
- 100mM DTT **Thermo Fisher Scientific Catalog #18090050**
- SuperScript™ IV Reverse Buffer (5X) **Thermo Fisher Scientific Catalog #18090050**
- Q5 Reaction Buffer (5X) **New England Biolabs Catalog #M0493S**

sparQ PureMag Beads **Quantabio Catalog #95196-060**

Nuclease-Free Water **Qiagen Catalog #129115**

Quick-RNA Viral 96 Kit **Zymo Research Catalog #R1041**

Agencourt SPRIPlate 96R Ring Super Magnet Plate **Beckman Coulter Catalog #A32782**

Zymo-Spin I-96 Plate **Zymo Research Catalog #C2004**

Nextera XT Index Kit v2 Set A (96 Indexes 384 Samples) **illumina Catalog #FC-131-2001**

10mM Tris-HCl (pH 8.0) **Contributed by users**

NextSeq500 v2.5 Mid-Output Kit **illumina Catalog #20024904**

IDT primers Custom ordered from IDT created by ARTIC Network v3 **IDT Catalog #Custom picked from ARTIC**

0.1 N NaOH **Contributed by users**

100% Ethanol **Sigma Aldrich Catalog #E7023**

RNA extraction

41m

1 Collect 96 patient samples and record sample data. Add 200 µL of the samples into each well of the Zymo-Spin™ I-96 plate. Keep plate On ice while preparing reagents in Step 2. 5m

2 Prepare the following reagents before starting viral RNA extraction: 2m
1. Add beta-mercaptoethanol (user supplied) to the Viral RNA Buffer to a final dilution of [M] 0.5 % volume i.e., 500 µL per 100 ml.
2. Add 192 mL of 100% ethanol (or 204 mL of 95% ethanol) to the 48 mL Viral Wash Buffer concentrate.

3 Add 400 µL of Viral RNA Buffer to each sample well. Mix well by pipetting. 2m

4 Mount the Zymo-Spin™ I-96 plate on a Collection Plate and centrifuge at 3000 x g - 5000 x g for 00:05:00. Discard the flow-through from the collection plate. 7m

5 Add 500 µL Viral Wash Buffer to each well and centrifuge at 3000 x g - 5000 x g for 00:05:00. Discard the flowthrough from the collection plate. Repeat this step. 12m

6 Add 500 µL ethanol (95-100%) to each well and centrifuge at 3000 x g - 5000 x g for 00:05:00. 6m

7 To elute RNA, mount a new 96-well plate between the Zymo-Spin™ I-96 plate and an empty Collection Plate. Add 15 µL DNase/RNase-Free Water directly to the column matrix of the Zymo-Spin™ I-96 plate wells and centrifuge at 3000 x g - 5000 x g for 00:05:00. The eluted RNA extract will be collected in the middle 96-well plate. 7m

cDNA preparation

1h 8m

8 Make a mastermix of the dNTPs and Random Hexamers for 96 samples (account for pipette error), pipette to mix and add 1 µL from mastermix to each well in a 96-well plate. 5m

To each well, add 5.5 µL of RNA extract eluted in Step 7.

Reagent	Amount
RNA Extract	5.5µl
10mM dNTPs Mix (NEB, N0446S)	0.5µl
50µM Random Hexamers (ThermoFisher, N8080127)	0.5µl
Total	6.5µl

9 6m

Set-up and run the following program on a thermal cycler to incubate reaction:

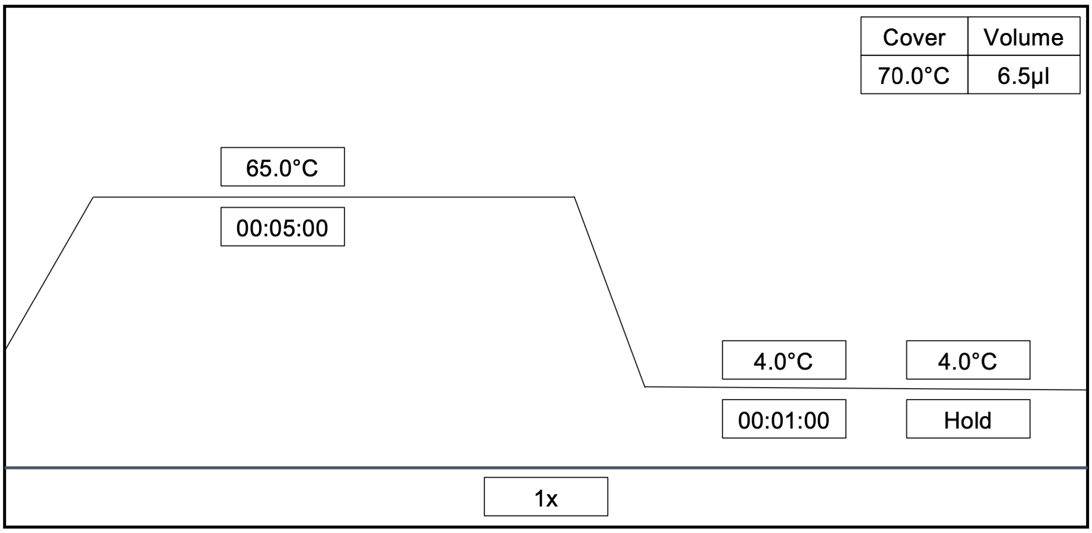

10 Make a mastermix of the following components for 96 samples (account for pipette error) and mix by pipetting. 5m

Add 3.5 µL of mastermix to each well with the annealed template RNA from Step 9.

Reagent	Amount
5X SuperScript™ IV Reverse Buffer (ThermoFisher, 18090050)	2.0µl
SuperScript™ IV Reverse Transcriptase (200U/µl) (ThermoFisher 18090050)	0.5µl
100mM DTT (ThermoFisher 18090050)	0.5µl
RNaseOUT Recombinant Ribonuclease Inhibitor (ThermoFisher, 10777-019)	0.5µl
Total	3.5µl

11 Set-up and run the following program on a thermal cycler to incubate reaction: 1h

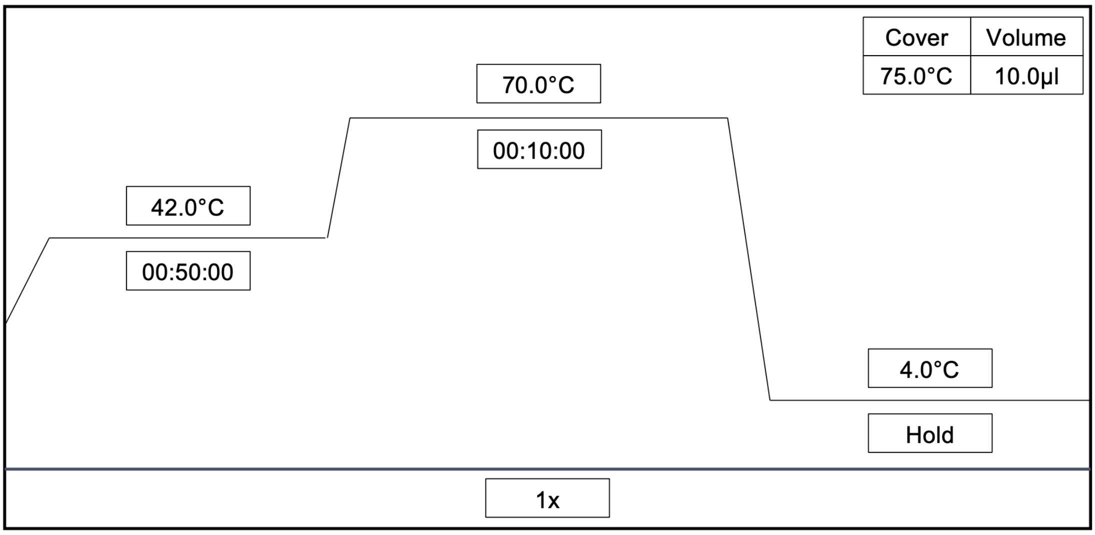

Multiplex PCR

8m

12 The following steps are performed in plates prepared according to the schematic below:

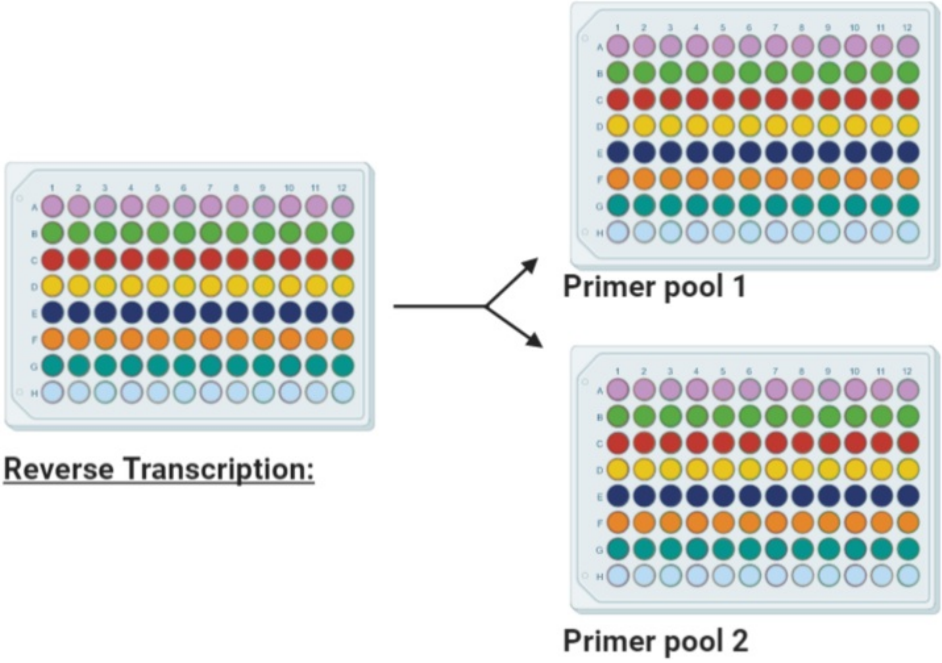

Two 96-well plates are prepared for PCR such that each contains a mastermix with the corresponding primer pool and cDNA from each biological sample.

13 Make a Pool #1 and Pool #2 master mix with the following reagents for 96 samples (account for pipette error) and mix by pipetting. In two 96-well plates, labeled Pool #1 or Pool #2, aliquot 22.5 µL of the corresponding Pool master mix into each well. 10m

Reagent	Pool #1	Pool #2
5X Q5 Reaction Buffer (NEB, M0493S)	5µl	5µl
10 mM dNTPs (NEB, N0446S)	0.5µl	0.5µl
Q5 Hot Start DNA Polymerase (NEB, M0493S)	0.25µl	0.25µl
10µM Primer (Pool 1 or 2)	3.6µl	3.6µl
Nuclease-free water (Qiagen, 129115)	13.15µl	13.15µl
Total	22.5µl	22.5µl

14 Add 2.5 µL of cDNA of each biological sample from from Step 11 to both Pool #1 and Pool #2 96-well plates. 2m

15 Set-up and run the following program on a thermal cycler to incubate reaction 5m 45s

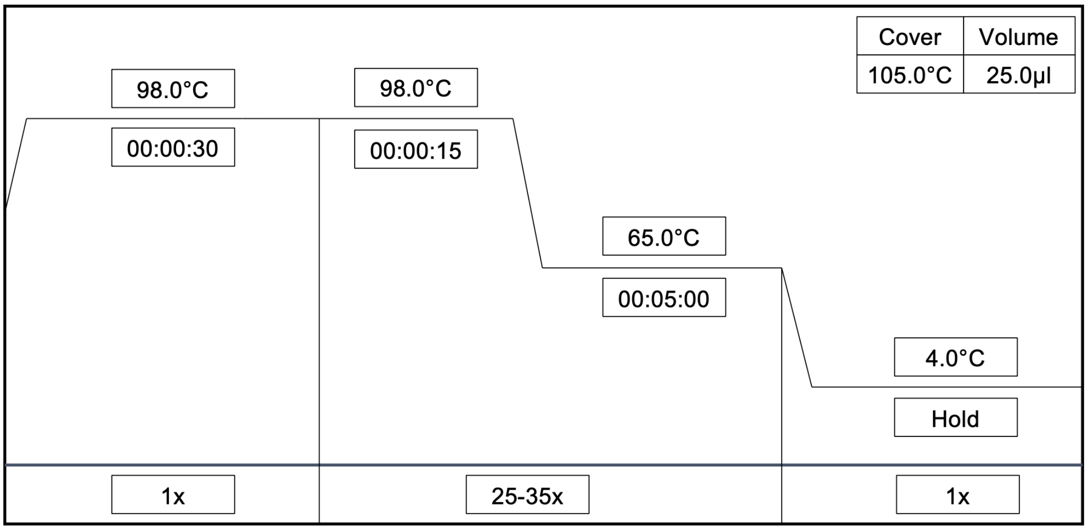

Post PCR Clean-up

15m

16 Add an equal volume (1:1) of sparQ PureMag beads to each sample well in plates Pool #1 and Pool #2 and mix gently by either flicking or pipetting. For example, add 50 µL sparQ PureMag beads to a 50 µL reaction. Then pulse centrifuge to collect all liquid at the bottom of the tube. 1m

17 Incubate for 00:05:00 at room temperature. 5m

18 Place the plates on the Agencourt SPRIPlate Super Magnet Plate and incubate for 00:02:00 or until the beads have pelleted and the supernatant is completely clear. Then, while avoiding the bead pellet, carefully remove the clear supernatant. 3m

Equipment	
SPRIPlate 96R Ring Super Magnet Plate	NAME
96-well Magnet Plate	TYPE
Agencourt	BRAND
A32782	SKU
https://www.beckman.com/supplies/plates/a32782	LINK

19 Keeping on magnetic plate, add 200 µL of Room temperature [M] 80 % volume (freshly made) ethanol to the side of the well opposite to the pellet and let sit for 00:00:30. 2m

20 Avoid disturbing the bead pellet, carefully remove and discard ethanol. Wait for 00:00:10 then remove any remaining ethanol. 50s

21 go to step #19 and repeat ethanol wash. 2m

22 Add 20 µL of [M] 10 millimolar (mM) Tris-HCl (Ph 8.0) and pipette to mix well. Incubate for 00:02:00 at 37 °C . 3m

23 Separate beads on the Agencourt SPRIPlate Super Magnet Plate for 00:02:00 or until the beads have pelleted. 3m

24 Transfer supernatant from each biological sample in the two plates (Pool #1 and Pool #2) and combine into a single well per sample in a new 96-well plate. 2m 30s

Quantification of DNA concentration using a Qubit® High Se... 1h

25 Quantify the DNA concentration using the Qubit® High Sensitivity DNA kit from 1 µL of each product using Steps 26-35.

26 Set up the required number of 0.5-mL tubes for standards and samples and label lids accordingly. 7m

27 Prepare a working solution by diluting the Qubit® dsDNA HS Reagent 1:200 in Qubit® dsDNA HS Buffer. For example, add 199 µL of buffer and 1 µL of dye per sample to a tube and vortex. 1m

28 Add 190 µL of Qubit® working solution and 10 µL of the standard to each of the standards tubes and vortex. 1m

29 For each sample, add 199 µL of Qubit® working solution and 1 µL of the sample in each 0.5-mL sample tube and vortex. 10m

30 Allow all tubes to incubate at room temperature for 00:02:00 2m

31 On the Home screen of the Qubit® 3.0 Fluorometer, press DNA, select dsDNA High Sensitivity, and press Read Standards to proceed. 2s

32 Insert the tube containing Standard #1 into the sample chamber, close the lid, then press Read standard. When the reading is complete (~ 00:00:03), remove Standard #1. 3s

33 Repeat Step 32 with Standard #2. 3s

34 To quantify samples, Press Run samples and select the sample volume (1 µL) and units. 3s

35 Repeat Step 34 for all samples. 6m

Nextera Library Preparation

36 Make a master mix of the Tagment mix and Tagment Buffer for 96 samples (account for pipette error) and pipette to mix well. Aliquot 6 µL of the master mix into each well of a new 96-well plate and add 4 µL of 1ng Amplicon DNA. 4m

Reagent	Amount
1ng of Amplicon DNA	4.0µl
Amplicon Tagment Mix (Illumina, FC-131-1096)	1.0µl
Tagment DNA Buffer (Illumina, FC-131-1096)	5.0µl
Total	10µl

37 Incubate at 55 °C for 00:05:00, then lower to 10 °C. Once at 10°C, immediately add 2.5 µL of Neutralizing Tagment Buffer to stop the reaction. Mix by pipetting up and down. 5m

38 Incubate at Room temperature for 00:05:00. Centrifuge at 280 x g for 00:01:00.

39 Make a mastermix of the Nextera PCR Master Mix and Index Primers 1 and 2 for 96 samples (account for pipette error) and mix by pipetting. Aliquot 12.5 µL of this mastermix into each well of a new 96-well plate and add 12.5 µL of tagmented amplicon DNA from Step 35. 1m

Reagent	Amount
Tagment Amplicon DNA	12.5µl
Nextera PCR Master Mix (FC-131-1096)	7.5µl
Index 1 primer (i7) (Illumina, FC-131-2001)	2.5µl
Index 2 primer (i5) (Illumina, FC-131-2001)	2.5µl
Total	25µl

40 Set-up and run the following program on a thermal cycler to incubate reaction: 9m 20s

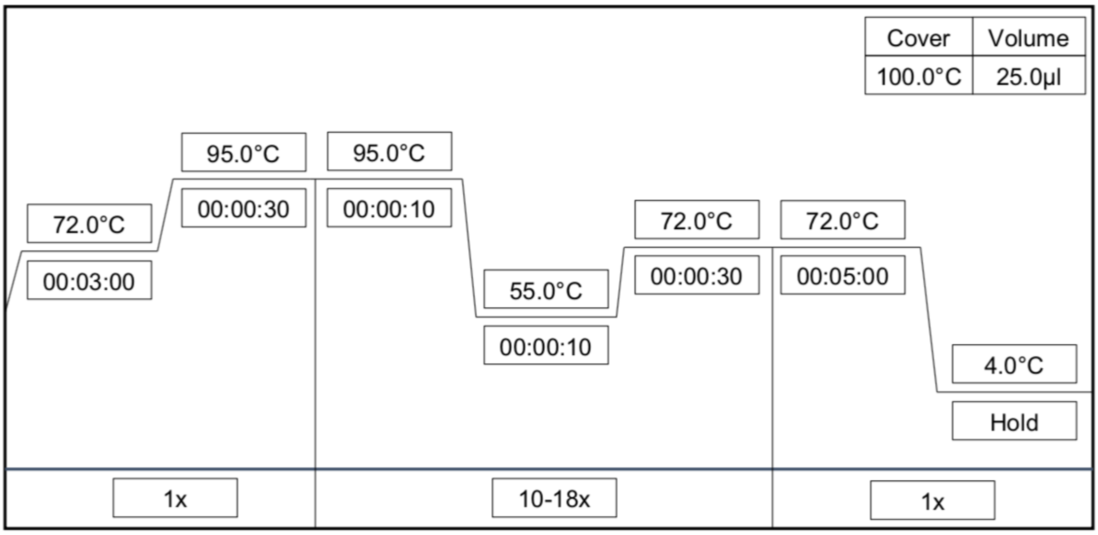

Final Library Cleanup

27m

41 After PCR, bring up sample volume in each well to 50 µL with nuclease free water.

42 Add a 1:1.8 ratio of sparQ PureMag beads to each well and mix gently pipetting. For example, add 90 µL sparQ PureMag beads to a 50 µL reaction. 30s

43 Incubate for 00:05:00 at room temperature. 5m

44 Place the plate on the Agencourt SPRIPlate Super Magnet Plate and incubate for 00:02:00 or until the beads have pelleted and the supernatant is completely clear. Then, while avoiding the bead pellet, carefully remove the clear supernatant. 2m

45 While keeping on magnetic plate, add 200 µL of Room temperature [M] 80 % volume (fresh made) ethanol to the side of the well opposite of the pellet and let sit for 00:00:30 . 1m

46 Avoid disturbing the bead pellet, carefully remove and discard ethanol. Wait for 00:00:10 then remove any remaining ethanol. 20s

47 Add 100 µL of [M] 10 millimolar (mM) Tris-HCl (pH 8.0) , vortex, and pulse centrifuge to collect all liquid at the bottom of the tube. Incubate for 00:02:00 at 37 °C . 2m

48 Separate beads on the Agencourt SPRIPlate Super Magnet Plate and transfer 100 µL of supernatant to a new plate for a second round of cleanup.

49 Add an equal volume (1:1) of sparQ PureMag beads to each well and mix gently by pipetting. For example add 100 µL sparQ PureMag beads to a 100 µL reaction. 30s

50 Incubate for 00:05:00 at room temperature. 5m

51 Separate beads on the Agencourt SPRIPlate Super Magnet Plate and incubate for 00:02:00 or until the beads have pelleted and the supernatant is completely clear. Then, while avoiding the bead pellet, carefully remove the clear supernatant. 2m

52 While keeping on magnetic plate, add 200 µL of Room temperature 1m

[M] 80 % volume (fresh made) ethanol to the side of the well opposite of the pellet and let sit for 00:00:30 .

53 Avoid disturbing the bead pellet, carefully remove and discard ethanol. Wait for 00:00:10 then remove any remaining ethanol. 10s

54 go to step #52 and repeat ethanol wash. 1m 30s

55 Add 20 µL of [M] 10 millimolar (mM) Tris-HCl (Ph 8.0) and mix well by pipetting. Incubate for 00:02:00 at 37 °C 2m

56 Place the plate on the Agencourt SPRIPlate Super Magnet Plate and incubate for 00:02:00 or until the beads have pelleted and the supernatant is completely clear. Then, while avoiding the bead pellet, transfer the 20 µL final library to a new plate for subsequent steps and storage. 2m

Library Normalization

30m

57 Transfer 10 µL supernatant from each well of the PCR plate to the corresponding well of a new midi plate.

58 Combine the following volumes in a 15 mL conical tube to prepare the LN master mix. Multiply each volume by the number of samples being processed: 23 µL of LNA1 and 4 µL of LNA2.

59 Pipette 10 times to mix and pour the LN master mix into a trough.

60 Use a p200 multichannel pipette to transfer 22.5 µL LN master mix to each sample well.

61 Seal the plate, and then use a plate shaker at 1800 rpm for 00:30:00

62 Place on the magnetic stand and wait until the liquid is clear (~2 minutes). Without disturbing the beads, discard all the supernatant.

63 Wash two times as follows:
Add 22.5 µL LNW1 to each well. Seal the plate, and then use a plate shaker at 1800 rpm for 00:05:00 . Separate on a magnetic plate and wait until the liquid is clear (~2 minutes). Without disturbing the beads, remove and discard all supernatant.

64 Add 15 µL 0.1 N NaOH to each well. Seal the plate, and then use a plate shaker at 1800 rpm for 00:05:00

65 Add 15 µL LNS1 to each well of a new 96-well PCR plate labeled SGP.

66 After the 00:05:00 elution completes, make sure that all samples in the midi plate are resuspended. If they are not, resuspend as follows:

66.1 Pipette 10 times to mix or lightly tap the sample plate on the bench. Seal the plate, and then use a plate shaker at 1800 rpm for 00:05:00 . Place on a magnet rack and transfer 15 µL supernatant from each well of the midi plate to thecorresponding well of the SGP plate.

66.2 Seal the plate, and then centrifuge at 1000 x g for 00:01:00 .

67 Place on a magnetic stand and wait until the liquid is clear ~ 00:02:00 .

68 Transfer 15 µL supernatant from each well of the midi plate to the corresponding well of the SGP plate.

69 Seal the sample plate, and then centrifuge at 1000 x g for 00:01:00 .

Loading the NextSeq550 sequencer

1h 20m

70 Remove a “Mid-Output Kit" reagent cartridge from -20°C freezer storage.

71 Place the cartridge right-side-up in a secondary container and fill the container with water at room temperature, but do not fully submerge the cartridge. Thaw cartridge in this water bath for about 01:00:00 .

72 When the reagent cartridge has been thawing for 00:30:00 , remove a "Mid-Output Kit" flow cell from the 4°C refrigerator but do not open the foil package. Place the flow cell on the bench for 00:30:00 to warm up to Room temperature

73 After the reagent cartridge is fully thawed, take it out of the water bath, tap on the bench to dislodge excess water, and dry with wipes.

74 Invert the cartridge to mix the reagents and make sure that the sequencing reagents in positions 29-32 are completely thawed. Then tap on the bench again to reduce air bubbles.

75 Remove the flow cell from its packaging and gently clean with a 70% ethanol wipe. Dry the glass with another wipe.

76 Inspect the flow cell to confirm: ports are free of obstructions, port gaskets are sealed and white plastic posts are visible, all 4 white retention clips are snapped over the edge of the black carrier plate and all 4 metal spring clips are laying flat against the black carrier plate.

77 Preheat an incubator to 98 °C

78 Remove a tube of Hybridization Buffer (HT1) from the -20°C freezer and thaw at Room temperature . When thawed, place on ice.

79 In a fresh 1.5ml tube, combine an aliquot from each library pool such that the total volume is 5 µL . For a Mid-Output kit, expect to obtain 150M reads in total.

80 Add 995 µL of ice-cold Hybridization Buffer (HT1). Vortex briefly and centrifuge at 300 x g for 00:01:00 .

81 Transfer 750 µL of diluted library to a new tube and add 750 µL of ice-cold Hybridization Buffer (HT1).

82 Vortex briefly and centrifuge at 300 x g for 00:01:00 .

83 Place the tube on the 98 °C heated incubator for 00:02:00 and immediately cool on ice. Leave on ice for 00:05:00

84 In a fresh 1.5ml tube, combine 1203 µL of ice-cold Hybridization Buffer (HT1) and 97 µL diluted library for a final concentration of [M] 1.5 picomolar (pM) . Invert to mix and pulse centrifuge. Place on ice until ready to load onto the reagent cartridge.

85 **Part 3: Load the Sequencer**

Using a clean P1000 tip, poke a hole in the foil seal covering position 10 on the reagent cartridge (labeled “Load Library Here”). Avoid breaking any of the other seals on the cartridge:

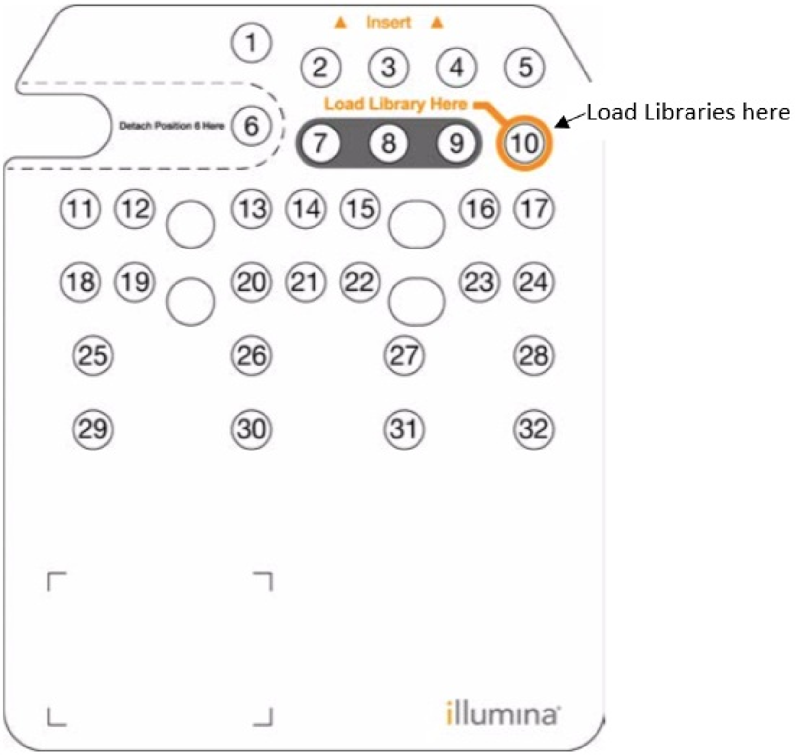

Finalized libraries are loaded in hole 10 of reagent cartridge.

86 Add the entire 1.3ml of the [M] 1.5 picomolar (pM) library dilution into this reservoir.

87 The reagent cartridge, flow cell, and buffer pack are now ready to be loaded onto the NextSeq500 for sequencing.
