# Supplementary material for: A rapid, low-cost, and highly sensitive SARS-CoV-2 diagnostic based on whole-genome sequencing
Source: PLoS One. 2023 Nov 30;18(11):e0294283. doi: 10.1371/journal.pone.0294283 (PMC10688730; doi:10.1371/journal.pone.0294283)
Supplement: S2 File — (DOCX) [file pone.0294283.s007.docx]

**Supplemental Text:**

*Calculation and comparisons of positivity threshold and limit of detection (LOD) from Calibration Data:*

POLAR’s positive result threshold was established empirically at genome coverage of >= 5%. This threshold yielded 95% accuracy for both positive and negative samples, as required by the FDA for SARS-CoV-2 test EUA. Having tested 20 (positive) samples at 84 GE/mL, 19 returned a breadth of coverage greater than 5%. Therefore, we could accurately classify 95% of samples tested at this concentration. We also tested 26 negative controls, of which 25/26 showed a breadth of coverage < 5%, thus allowing us to accurately classify > 95% of negative samples.

Another approach we used was to perform a linear regression on the approximately linear portion of the “calibration curve” (concentrations 0, 84, and 840 GE/mL). The resulting linear equation was %coverage = 0.059*concentration [GE/mL] + 6.8%. The Y-intercept of the concentration-coverage curve (i.e. where concentration = 0 GE/mL) would be at 6.8%. Utilizing this threshold would have led to 26/26 correctly identified negatives, and 18/20 correctly identified positives. Given our thinking that a false negative would be more harmful than a false positive, we erred on the side of slightly lowering that threshold.

While the FDA defined the LOD as the lowest concentration (GE/mL) that would allow the identification of 19/20 positives and negatives, we can also utilize the LOD equation LOD = 3.3·σ/S where σ is the standard deviation of measurements where the independent variable is zero, and S is the slope of a curve fit to the data. As above, we make an assumption that the low-concentration portion of the “calibration curve” is linear; this relatively linear portion of the curve was utilized to compute the LOD. The above expression assumes homoscedasticity, which we confirm with the Breusch-Pagan test (p >> 0.05, thus not rejecting the null hypothesis of homoscedasticity). The calculated LOD is 87 GE/mL, which is very close to our empirically identified one (84 GE/mL).

LOD is the metric we utilized as a quality measure for the test. This approach seemed reasonable given the LOD’s relationship to sensitivity and specificity - assuming viral concentration in the wild is arbitrarily dilute, being able to accurately discern positives from negatives at low concentrations will increase both sensitivity and specificity. LOD is also a metric uniformly reported across tests. Thus per the FDA EUA standards, “sensitivity” is inversely correlated to LOD (being able to detect lower concentrations will allow for a higher rate of detection of ‘true positives’ given ‘true positives’ could be arbitrarily dilute).

We realize the FDA’s requirement is a sensitivity as typically defined (true positives/(true positive + false negatives) at particular concentration ie true positives/(true positive + false negatives) >= 95% at a specific concentration of virus. A test’s LOD is the lowest level at which this sensitivity is reached.

Time requirements for the test were not routinely reported hence comprehensive comparisons were not possible. Additionally, some methods with EUA operate across several instrumentation models for automation and/or real-time PCR machines. This led to single approved tests having varied performance times.
